# Formal Analysis of the Security Protocol with Timestamp Using SPIN

**DOI:** 10.1155/2022/2420590

**Published:** 2022-08-23

**Authors:** Meihua Xiao, Weiwei Song, Ke Yang, Ri OuYang, Hanyu Zhao

**Affiliations:** School of Software, East China Jiaotong University, Nanchang 330013, China

## Abstract

The verification of security protocols is an important basis for network security. Now, some security protocols add timestamps to messages to defend against replay attacks by network intruders. Therefore, verifying the security properties of protocols with timestamps is of great significance to ensure network security. However, previous formal analysis method of such protocols often extracted timestamps into random numbers in order to simplify the model before modeling and verification, which probably cause time-dependent security properties that are ignored. To solve this problem, a method for verifying security protocols with timestamps using model checking technique is proposed in this paper. To preserve the time-dependent properties of the protocol, Promela (process meta language) is utilized to define global clock representing the protocol system time, timer representing message transmission time, and the clock function representing the passage of time; in addition, a mechanism for checking timestamps in messages is built using Promela. To mitigate state space explosion in model checking, we propose a vulnerable channel priority method of using Promela to build intruder model. We take the famous WMF protocol as an example by modeling it with Promela and verifying it with model checker SPIN (Simple Promela Interpreter), and we have successfully found two attacks in the protocol. The results of our work can make some security schemes based on WMF protocol used in the Internet of things or other fields get security alerts. The results also show that our method is effective, and it can provide a direction for the analysis of other security protocols with timestamp in many fields.

## 1. Introduction

With the development of the industrial Internet of things, 5G, blockchain, and cloud computing, many related security issues [1, 2] have arisen at the same time. As an important basis for security in these fields, security protocols are now attracting more and more researchers' interest. The main purpose of using a security protocol is to ensure the security of network communication, but the protocol itself is vulnerable to attacks by network intruders. A seemingly simple and correct security protocol may have vulnerabilities; a typical example is the Needham-Schroeder public key protocol, whose simplified version has only three exchanged messages, but it was discovered by Lowe [3] 17 years after its publication that there is a security vulnerability, which allows intruders to destroy the authentication of the protocol. Because security protocols are difficult to check intuitively and analyzing the security of protocols by relying on empirical principles is inefficient, currently the most effective way to analyze and verify security protocols is formal methods [4]. Model checking [5] is one of the formal verification methods, and SPIN [6], as a simple and efficient model checker, has been widely used in the field of protocol verification. Reference [7] proposed a method of statically analyzing the knowledge of the intruder and then using SPIN to formally verify the classical Needham-Schroeder public key authentication protocol and successfully find the vulnerability. After that, some scholars made further research on the basis of this method and used SPIN to analyze and verify the MANET protocol [8], the RFID three-party authentication protocol [9], and the OAuth 2.0 protocol [10], respectively. Reference [11] proposed a method for formal analysis and verification of the authentication and secrecy of a class of security protocols using SPIN/Promela. In that paper, the intruder can dynamically intercept message in the network, which help the intruder perform replay attacks or forgery attacks on security protocols. The intruder model of this method is efficient but the number of state transitions is large; if this method is used to analyze security protocols with timestamps, the problem of state space explosion is prone to occur. The methods in [12, 13] alleviated the problem of state spaces explosion, but they aimed at security protocols without timestamps. The above methods can use SPIN to analyze and verify the authentication or secrecy of security protocols with random numbers, but the properties of security protocols with timestamps [14–18] are closely related to specific time factors, so the above methods are difficult to work for them. In fact, the formal analysis of security protocols with timestamps is difficult, and the difficulties are mainly reflected in two aspects: First, the security protocols with timestamps are applied in real-time systems, and not only the protocol agents but also a time model to describe various time factors in the protocol needs to be modeled during analysis, so the overall model is more complex and prone to state explosion problems; second, it is difficult to convert time-dependent properties into formulas or languages that can be recognized by the verification model. Therefore, timestamps were often extracted into random numbers which probably caused time-dependent security properties that are ignored in the previous work of formal analysis of security protocols with timestamps. Aiming at the above problems, this paper proposes a modeling method that preserves the time factors of the protocol by using the Promela assertion that SPIN can recognize to express the time-dependent property, so that the freshness of protocol message can be verified. We take the well-known WMF (wide-mouth frog) protocol [19] that plays an important role in some IoT security scenarios [20, 21], which contains timestamps as an example to illustrate our method. By applying our method, one attack path [22, 23] that violates key freshness and another that violates the authentication in the WMF protocol are successfully found.

The main contributions of this paper are as follows:

1. A modeling method that preserves the time factors of the protocol with timestamp is proposed
2. A way of expressing key freshness property that can be converted into Promela assertions is proposed
3. We propose a method of using Promela to build intruder model based on vulnerable channel priority to mitigate state space explosion and successfully find the attacks of WMF protocol

The structure of this paper is arranged as follows. In the next section, we give the overall scheme for analyzing security protocol with timestamp using SPIN. In Section 3, preliminary knowledge about WMF protocol is given. We dedicate Section 4 to use Promela to model the WMF protocol. In Section 5, we use assertion and linear temporal logic (LTL) to indicate key freshness and authentication property of WMF protocol, respectively. In Section 6, we present the experiment result with SPIN. We conclude in Section 7 by a summary and outlook.

## 2. The Overall Scheme

**Figure 1:**
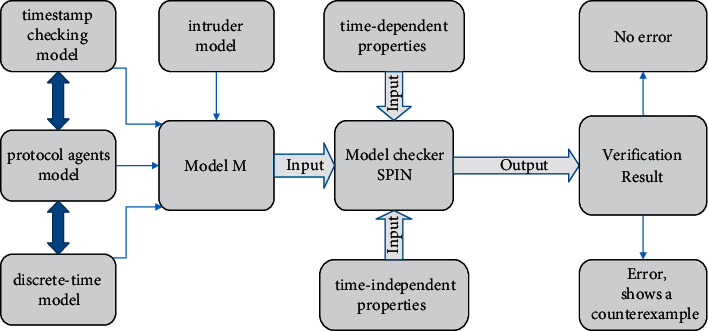
Security protocol with timestamp verification scheme.

In this section, we give the overall scheme which shows all the modeling and verification work of this paper and the overall analysis diagram is shown in Figure 1. Our scheme is divided into three steps as follows:

 
*Step 1.* Use Promela to build a complete model M, which includes four model parts: discrete-time model part, protocol agent model part, timestamp checking model part, and intruder model part.
 
*Step 2.* Use assertion to represent time-dependent properties, such as key freshness, and use LTL represent other time-independent properties, such as authentication.
 
*Step 3.* Input the above model and security properties into model checker SPIN to automatically verify whether the protocol model satisfies the properties.

Note that the verification process is automated because of the excellent algorithm design within SPIN and if the protocol does not satisfy the security property, SPIN will generate a counterexample and give the specific attack path of the counterexample.

In fact, each step above corresponds to one of the third to fifth sections where you can see the details of the analysis process of the WMF protocol. However, we must introduce some preliminary knowledge about the WMF protocol in next section before we analyze the protocol.

## 3. WMF Protocol

### 3.1. The Description of WMF Protocol

The WMF protocol is an authentication and key agreement protocol which was specially designed to provide secure data transmission and authentication services to insecure networks. The protocol can be described as follows:

(1) $$\begin{array}{c} A->S:A,\left\{T_{A},B,K\_AB\right\}K{}_{AS},\\ S->B:\left\{T_{S},A,K\_AB\right\}K{}_{BS}. \end{array}$$

As you see above, the protocol is divided into two steps.

 
*Step 1.* Agent *A* as an initiator is responsible for generating a temporary session key *K_AB* shared with *B*, encrypting it together with *B*'s agent identifier and A's current time *T_A_* to form a ciphertext block {*T_A_, B, K_AB*} *K_AS_*. Then *A* attaches its own agent identifier *A* to form Message1: *A*, {*T_A_, B, K_AB*} *KAS* and send it to the trusted server *S*.
 
*Step 2.S* is responsible for encrypting the key *K_AB* and the agent identifier of *A* in the received message with the server's current time to form *Message2*: *{Ts, A, K_AB} K_BS_,* and send it to *B.*

It should be noted that when the server *S* receives the message sent by agent *A*, it will check the timestamp *T*_*A*_ in the message to determine whether the message is fresh. When agent *B* receives a message from server *S*, it will check whether the timestamp *Ts* in the message is newer than the timestamp sent by *S* before (to resist replay attacks). If *Ts* is the latest, the message will be determined by *B* that is fresh. And *B* determines the initiator of the protocol based on the agent identifier in the message to complete one-way authentication.

### 3.2. Security Properties of WMF Protocol

Two fundamental security properties of the WMF protocol are key freshness and authentication. The key freshness requires that the session key received by the receiver must be generated by the current round of protocol sessions. The authentication, which includes one-way authentication and two-way authentication, is used to determine whether the identity of the communicating party is consistent with the identity claimed in the message. What the WMF protocol needs to satisfy is the one-way authentication. In order to strictly describe the key freshness and one-way authentication, some basic symbols are first defined. The specific symbols and their meanings are shown in Table 1. Based on the basic notation, the definitions of key freshness and one-way authentication are given below.

**Definition 1 .**

(*key freshness*). Assuming that the agent's processing of messages is completed immediately, protocol *P* satisfies *key freshness* if and only if

(2) $$\begin{array}{c} N*D\text{max}\geq Tn-Ts\&\&N*D\text{min}\leq Tn-Ts. \end{array}$$

**Definition 2 .**

(*one-way authentication*). The protocol *P* satisfies the *one-way authentication,* if and only if *Rec* confirms its receipt or indirect receipt via *Ser* message from *Init* after *Init* initiates or indirectly initiates via *Ser* a session with *Rec.*

### 3.3. Intruder Rule Description

For the intruder, this paper adopts the Dolev-Yao [24] intruder model specification. In this model specification, an intruder has complete control over the network, and the intruder has the following capabilities:

1. Ability to eavesdrop, block, and intercept all messages on the network
2. Ability to send and resend messages
3. Decompose and combine messages
4. Ability to impersonate any protocol participant
5. After knowing the decryption key, the intruder can decrypt the encrypted message
6. Familiarize all the agent identifiers participating in the protocol

Although the intruders are powerful, they are not omnipotent. For example, the intruder cannot decrypt the ciphertext without the corresponding key, and the intruder cannot infer the key according to the ciphertext. The model specification assumes that the cryptographic algorithm used by the protocol is perfect, which is beneficial for us to focus on the protocol design level without caring about the cryptographic system.

**Figure 2:**
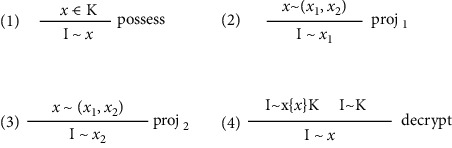
Knowledge deconstruction rules.

Although intruders can intercept messages from the network, they need to follow certain rules, which can be used to constrain the Promela model of the intruder in Section 4.4, to decompose and combine messages [11]. After an intruder gets a message and wants to learn knowledge from it, he needs to follow the rules in Figure 2, where I ∼ *x* indicates that the intruder can deduce and get *x*. Rule 1 means that if the intruder possesses some knowledge, he can deduce the same knowledge in the message; Rule 2 and Rule 3 state that if the intruder can get some knowledge, he can deduce the part of knowledge in the message; Rule 4 means that if the intruder can get both the encrypted message component *{x}K* and the key *K*, then he can decrypt the message component and gain knowledge.

**Figure 3:**
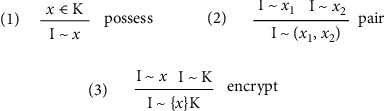
Knowledge construction rules.

Meanwhile, the intruder can create new messages based on their knowledge. The creation of new messages needs to follow the construction rules of Figure 3.

Rule 1 means that if the intruder has some knowledge, he can create an integral message based on this knowledge; Rule 2 indicates that if the intruder can get some integral knowledge, he can create message components which is part of integral knowledge; Rule 3 means that if the intruder can get the knowledge *x* and the corresponding key *K*, it can create encrypted message components {*x*}*K*.

## 4. WMF Protocol Model

Promela (process meta language) [5] is a modeling language for describing concurrent systems, and it is also the input language for the model checker SPIN. Promela uses processes to represent system behaviors and achieves information exchange between different processes through message channels. Promela can simulate the behavior of protocol agents and the communication between protocol agents, so it is very suitable for modeling security protocols.

In this section we take the WMF protocol as an example to illustrate the entire process of building the security protocol model with timestamps. The WMF protocol is modeled using Promela language, including four parts: discrete-time model, timestamp checking model, protocol agent model, and intruder model. It should be noted that the model is based on the following assumptions, which prevent other factors from affecting the protocol model.

**Assumption 1 .**

The receiver trusts the initiator to be capable of generating a perfect key.

**Assumption 2 .**

The clocks between different agent and servers in the protocol runtime environment are synchronized.

**Assumption 3 .**

Intruders cannot tamper with the clock, and all timestamps in the protocol indicate the real current time.

### 4.1. Discrete-Time Model

**Figure 4:**
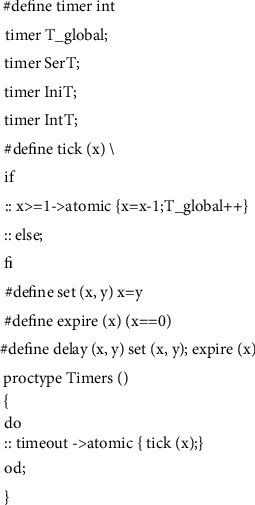
Discrete-time model.

Security protocols with timestamps not only need to consider the time order of process communication, but also need to consider the specific time spent in communication, so we build a discrete-time model as shown in Figure 4. The first macro *timer* is used as an alias for *int*, which is used to represent discrete-time type. The second macro *tick* (*x*) represents the passage of time, and the parameter *x* is a timer variable. Each time the tick statement is executed, the value of *x* is reduced by one, which means that a unit of time has passed. At the same time, the value of the global clock variable *T_global* increases by one, which means one unit of time of the system elapses. *T_global* also plays the role of clock synchronization in the system because the current time of all agents in the model is represented by it. The value of the *T_global* will change with the elapse of time caused by any agent transmitting a message. The third macro *set (x, y)* is used to assign a value represented by *y* which indicates message transmission time to the timer variable *x*. The fourth macro *expire(x)* is a blocking statement stopping the program, and only if *x* is equal to 0, which indicates that the message has been transmitted in the channel, the program will continue to execute at this time; instead, the process in which the statement is located will be blocked if *x* is not equal to 0, which means there are still messages in transit. The *timeout* is a predefined Boolean variable in Promela. The value of *timeout* is *true* when all other processes in the system enter the blocking state, and in this situation the *tick* statement is executed to simulate the passage of time. The *Timer* process works with *set (x, y)* and *expire(x)* to model the protocol transmission time. For example, if *statement_sent* and *statement_rec* represent message sending and message receiving in the protocol model, respectively, then *{statement_sent; set(x,5); expire(x); statement_rec}* statement indicates that the message receiving statement will be executed after 5 units of time after the message sending statement is executed. Therefore, *set (x, y)* and *expire(x)* are combined into the fifth macro *delay (x, y)* to directly represent the transmission time of the message.

### 4.2. Timestamp Checking Model

**Figure 5:**
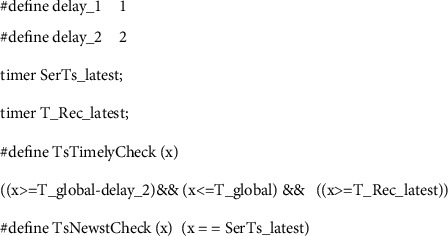
Timestamp checking model.

We define two functions *TsTimelyCheck(x)* and *TsNewstCheck(x)* to achieve the checking of timestamps by the server and the receiver, respectively. In the WMF protocol, to ensure that the message is fresh, the server not only determines whether the timestamp in the received message from an agent is newer than the timestamp sent by the agent before, but also determines whether the timestamp in the received message is within the allowed time window. The receiver will decide whether the timestamp in the received message is newer than the timestamp sent by the server before, and the receiver will accept the message if it receives the latest timestamp. Therefore, the timestamp checking model of the server and receiver is shown in Figure 5. Of course, the model will be embedded in the server process and the receiver process in the following section.

### 4.3. Protocol Model

The first step in modeling the agent of the protocol is to construct a set of variable names. We define a set of names representing all possible variables (except timestamps) in WMF protocol, including honest agent *A*, agent *B*, server *S* and intruder *I*, and all possible keys *Kab, Kas, Kbs, Kis, Kbi, Kai*. But the timestamp is not placed in the variable names set, because the timestamp variable is part of the time model. Set of variable names for the WMF protocol is as follows:

(3) $$\begin{array}{c} m\text{type}=\left\{A,B,I,S,Kab,Kas,Kbs,Kis,Kbi,Kai\right\}. \end{array}$$

The second step is to model the message channel through which the communication takes place. There are two messages in the WMF protocol, and different messages correspond to different message structures, so we define two synchronization channels, each of which is responsible for transmitting messages of one structure. Promela model for two synchronization channels is as follows:

(4) $$\begin{array}{c} \text{chan}c1=\left[0\right]\text{of}\left\{m\text{type},\text{timer},m\text{type},m\text{type},m\text{type}\right\};\\ \text{chan}c2=\left[0\right]\text{of}\left\{\text{timer},m\text{type},m\text{type},m\text{type}\right\}. \end{array}$$

In there, *mtype* represents the enumeration type, and its value range is all the variables in the variable name set constructed in the first step, which are used to represent all other types of variables in the protocol message except the timestamp. In messages containing timestamps, the timer type variable is used to represent the timestamp. For example, timer *T*_*A*_ = 2, indicating that the value 2 of the timestamp *T*_*A*_ in the message is the time when the message was sent. The third step is the realization of the protocol role. An honest agent in the WMF protocol can play roles as initiator, server, and receiver, and each role corresponds to a process in our model.

**Figure 6:**
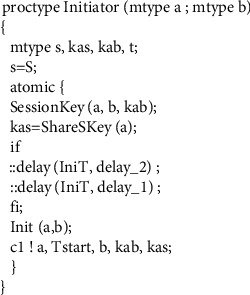
Process of initiator.

**Figure 7:**
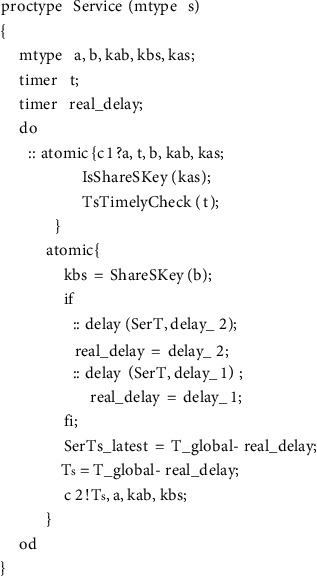
Process of server.

**Figure 8:**
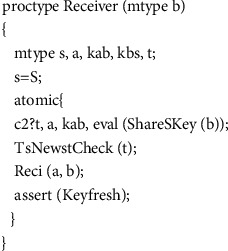
Process of receiver.

**Figure 9:**
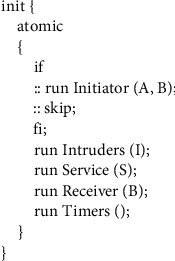
Process of role instantiation.

(1) The realization of initiator process is as shown in Figure 6.
The initiator role process has two parameters representing the initiator itself and the agent for which it wants to establish a temporary session key. In the macro *Sessionkey (a, b, kab)*, the parameters *a* and *b* represent two agents that want to communicate with each other, and the parameter *kab* represents the session key for secret communication between agent *a* and agent *b*. According to the pairwise combination of agents *A*, *B*, and *I*, there are three possible session keys in total as follows:
  (5) $$\begin{array}{c} \#\text{defineSessionKey}\left(a,b,k\right)\text{if}\\ \text{::}\left.\left(\left(a==A\right)\right.\&\&\left(b==B\right)\right)\|\left(\left(a==B\right)\&\&\left(b==A\right)\right)->k=Kab\\ \text{::}\left.\left(\left(a==I\right)\right.\&\&\left(b==B\right)\right)\|\left(\left(a==B\right)\&\&\left(b==I\right)\right)->k=Kbi\\ \text{::}\left.\left(\left(a==A\right)\right.\&\&\left(b==I\right)\right)\|\left(\left(a==I\right)\&\&\left(b==A\right)\right)->k=Kai\\ \text{fi} \end{array}$$
The full definition of the macro *Init (a, b)* is
  (6) $$\begin{array}{c} \text{define Init}\left(a,b\right)\text{ if::}\left(a==A\&\&b==B\right)->InitAB=1\text{;::else;fi}. \end{array}$$
The specific meaning of it is that the initiator A initiates a protocol session for key establishment with the receiver *B*. *ShareSKey(a)* represents the shared key between agent *A* and server *S*. The *delay* statement is used to indicate the transmission time from the initiator sending the message to the server receiving the message. In order to be able to validate models using both asynchronous and synchronous channels without changing the order of statements, we place the *delay* statement before the sending statement, but this does not cause an error in the time model in the protocol, because in a synchronous channel in Promela, sending a message and receiving a message happen at the same time. For example, if A is a sending statement in one process and B is a receiving statement in another process, then for the entire system, the state after executing *atomic {delay (x, y); A; B}* and the state after executing *atomic {A; delay (x, y); B}* are completely equivalent. In Section 6 we will present two experimental results, one for asynchronous channels and another for synchronous channels.
The body of the *if* statement in Figure 6 is a selection structure. The statement marked with “::” in this structure indicates that the statement will be executed randomly; that is, only one of the multiple *delay* statements in the *if* will be executed randomly. This can be done by doing the simulation of random transit time.
(2) The realization of the server role is shown in Figure 7.
In the server role process, *IsShareSKey(kas)* is statement whose function is to decide whether the parameter *kas* is a key shared with the server, so as to identify whether the message is sent to the server. *TsTimelyCheck(t)* is used to check whether the received message is fresh. *Atomic* is a reserved word in the Promela language, and the statements in the program block identified by *atomic* are not executed in cross-execution with the statements in other processes even in a concurrent environment but are executed in a step-by-step sequence. This mechanism can play a role in state compression, thereby alleviating the problem of state space explosion.
(3) Then comes the receiver role realization, as shown in Figure 8.
In Figure 8, *eval* is a matching function predefined by Promela, which is used to check whether the value sent from the channel is equal to the value in the parameter. If it is equal, the message is received and if it is not equal, it is not received. *eval(ShareSKey(b))* indicates that the key used to encrypt the message on the channel must be the shared key between the server and the receiver. This checking ensures that messages on the channel can only be received by the specified agent. *TsNewstCheck(t)* is used to check whether the timestamp in the message is the latest timestamp generated by the server. If it is the latest, it means that the message is within the valid time range. *Reci(a, b)* means that the receiver confirms that it has received the key sent by the initiator. The *assert* statement, which determines whether the value of its parameter is true, is used here to verify the freshness of the key in the received message.
(4) The fourth step is instantiation of roles, as shown in Figure 9.

In Figure 9, the *init* process is the main process of Promela. When the system starts, the *init* process will be executed first, which is equivalent to the *main* function in the C language. But before the system starts, the parameters of the process are assigned by real agent in the protocol and keyword *run* is used to activate each role process and the *Timers* process representing the time model in the system. So far, the entire protocol model has been built. The next step is to build the intruder model and put it into the protocol model to simulate the real insecure network environment.

### 4.4. Intruder Model

In model checking, the number of states increases exponentially with model complexity. If the traditional dynamic analysis method is used to analyze all possible knowledge of the intruder, the problem of state explosion is prone to occur because our model has more time factors than the general model. Therefore, the intruder model must be adjusted to avoid the problem.

**Figure 10:**
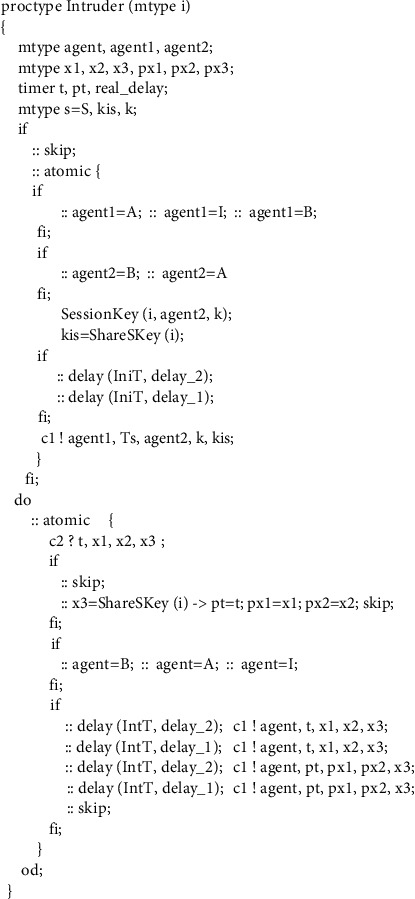
Process of intruder.

Our solution is to refine the intruder's capabilities by using vulnerable channel priority, which is a simple but useful modeling method. The method includes two steps. The first step is to manually analyze and find out the vulnerable channel, that is, the channel where it is easier for the intruder to implement the intrusion. The second step is to model the intruder according to the identified vulnerable channels. It should be noted that we must gradually increase intruder capabilities in our model if no vulnerabilities are found until an attack is found or a state space explosion occurs. Compared with the method that intruders dynamically intercept messages on all channels, this modeling method can greatly reduce the number of state transitions in the process of model checking because the intruder capability in this method is not so strong at first. We take the WMF protocol as an example to describe the method. In WMF protocol, we discard the intruder's ability to intercept messages on the first channel, in which the initiator sends message to server, and retain the ability to intercept messages on the second channel. The reason is that, in practice, the server checks the timestamp much more strictly than the receiver and the intruder prefers to start to attack from places with lower security defenses. So, we chose the second channel as the vulnerable channel in the WMF protocol through simple analysis. Fortunately, we quickly found WMF protocol attacks using the vulnerable channel priority intruder model building method. The intruder's Promela model is shown in Figure 10.

We divide the intruder's capability model into two parts. The first part corresponds to the outermost *if* structure in Figure 10, and its function is to send any possible message into the channel using its own initial knowledge. The second part corresponds to the *do* loop structure in Figure 10. Its function is to make the intruder intercept and utilize messages in the communication channel. The intruder's ability to intercept and process messages is a loop operation that will not actively stop, so as to maintain constantly intercepting messages on the network so that the intruder can learn more knowledge. In addition, after intercepting the message, if the intruder has the key of the message, he can learn and store the knowledge in the message, so as to use these messages to replay or forge new messages at an appropriate time. If the intruder cannot decrypt the encrypted component in the message, the entire encrypted component can be forwarded to the channel or nothing can be done.

After the intruder model is established, the next section needs to put the protocol's security specification into the model to verify whether the security properties of the protocol are still satisfied in the network environment where the intruder exists.

## 5. Security Properties

The two security properties that the WMF protocol needs to satisfy are freshness and authentication. Freshness requires that the message received by the recipient must be fresh, which contains two meanings: the first meaning is that the message must be generated recently, which can be guaranteed by the timestamp checking mechanism in the model, so no verification is required. The second meaning is that the session key received by the receiver must be the fresh key generated by the current round of the protocol session, and we use an assertion to verify this property. Promela assertion is a function in the format *assert(expression)*, whose function is to determine whether the value of the *expression* is true. If false, SPIN will report an error and give a counterexample.

According to the definition of security property in Section 3.2, *assertion* can be used to verify the key freshness in WMF protocol as *assert (Keyfresh)* where the logical expression *Keyfresh* is completely defined as follows:

(7) $$\begin{array}{c} \text{\#defineKeyfresh}\left(\left(\text{T\_global}-\text{Tstart}\right)<=2*\text{delay\_}2\right)\text{\&\&}\left(\left(\text{T\_global}-\text{Tstart}\right)>=2*\text{delay\_}1\right). \end{array}$$

In this expression, *delay_1* and *delay_2* represent the minimum and maximum time consumed by the communication channel to transmit a message, respectively. The number *2*, which is equivalent to the value of *N* in Section 3.2, indicates that one round of WMF protocol has two messages containing the key transmitted through the channel. *Tstart* indicates the key generation time, which is also the time when the initiator initiates the session. *T_global* is the global clock variable, which is used here to indicate the moment when the message was received.

This assertion indicates that the time elapsed from the time the key is generated to the time it is received by the receiver must be within a reasonable transmission time range; otherwise the key is not a valid fresh key.

The WMF protocol also needs to complete the one-way authentication of the key receiving agent to the key generating agent. In order to use LTL to represent the authentication of the WMF protocol, we must define the following variables.

bool *InitAB* = 0, set to 1 when the agent *A* sends a message to the message channel;

bool *ReceAB* = 0, set to 1 when the agent *B* confirms receipt of the message from *A*. The LTL formula which indicates the authentication based on the above variables is as follows:

(8) $$\begin{array}{c} \left(\left[\right]\left(\left[\right]\text{!Reci}AB\|\left(\text{!ReciAB }U\text{ InitAB}\right)\right)\right). \end{array}$$

This formula means that the receiver *B* can never acknowledge receipt of a message from initiator *A* until *A* initiates a session with *B*.

The assertion representing the key freshness and the LTL formula representing the authentication with the Promela model of the WMF protocol in Section 4 of this paper are imported into the model checking tool SPIN, and SPIN can automatically verify whether the protocol satisfies the security properties.

## 6. Experimental Results

**Figure 11:**
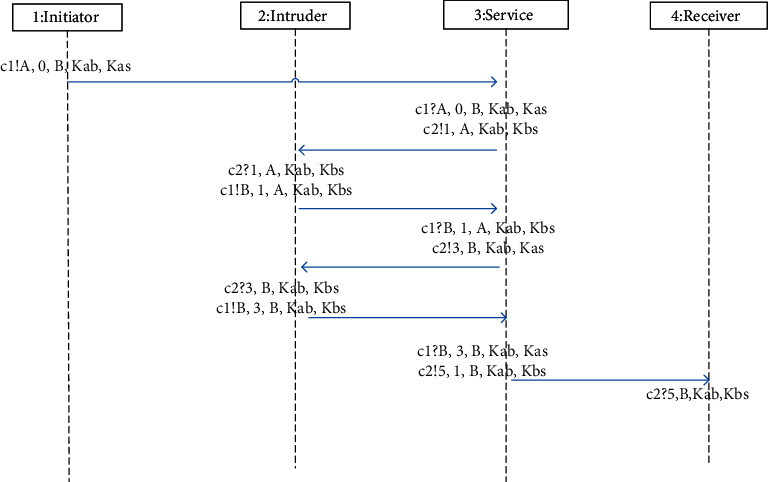
Attack path of WMF protocol violating key freshness.

**Figure 12:**
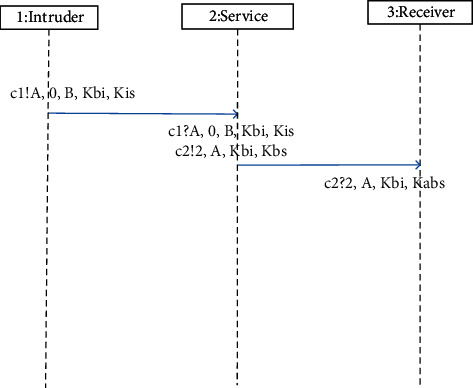
Attack path of WMF protocol violating authentication.

In the Promela model of WMF protocol (called Model 1) established in Section 4, different processes communicate through synchronization channels. However, in practical applications, asynchronous channels are often used for communication between different processes. Therefore, we changed the message channel in Model 1 to an asynchronous channel with a channel capacity of 1, and the other parts remained unchanged to obtain a new Promela model (called Model 2). Using SPIN to verify these two models, the experimental results show that the models using these two message channels can successfully find the attack paths, which are shown in Figures 11 and Figures 12, of the counterexamples that are the same. The experimental data are shown in Table 2. The number of stored states can reflect the complexity of the model; the number of state transitions can reflect the efficiency of state search in model checking.

As can be seen from Table 2, our models have no state space explosion, and the number of stored states and state transitions is small, which is mainly due to the experimental strategy of vulnerable channel priority.

Table 3 shows the results of using different methods to find the attack path of the WMF protocol. It can be seen from the table that the methods of [22] and [23] can only find the attack path 1 of the WMF protocol, but the method in this paper can find both the attack path 1 and the attack path 2. We must state that an important premise of the attack is that the server in actual implementation of the protocol can infer the key, which is owned by the server, to the ciphertext from the message format.

Figure 11 shows the attack path of WMF protocol violating freshness (attack path 1). We denote by *I(X)* that the intruder *I* impersonates the agent *X*; then, the attack path of violating key freshness is summarized as follows:

(9) $$\begin{array}{c} A->S\text{:}A,\left\{T_{A},B,K\_AB\right\}K_{AS}. \end{array}$$

Agent *A* sends a message with the key *K_AB* to server *S*;

(10) $$\begin{array}{c} S->I\left(B\right)\text{:}\left\{T_{S},A,K\_AB\right\}K_{BS}. \end{array}$$

After the server *S* receives the message, it updates the timestamp and sends the message containing *K_AB* to the agent *B* with the identifier of *A*, but the message is intercepted by the intruder *I*;

(11) $$\begin{array}{c} I\left(B\right)->S\text{:}B,\left\{T_{S},A,K\_AB\right\}K{}_{BS}. \end{array}$$

The intruder *I* pretends to be *B*, and sends the encrypted message that was intercepted last time with the identifier of *B* to the server *S*;

(12) $$\begin{array}{c} S->I\left(A\right)\text{:}\left\{T_{S}^{\prime },B,K\_AB\right\}K{}_{AS}. \end{array}$$

*S* believes that the received message is a new protocol session message sent by agent *B*, so it adds the new timestamp into the message and sends the message to agent *A*, but the intruder *I* intercepts the message again;

(13) $$\begin{array}{c} I\left(A\right)->S\text{:}A,\left\{T_{S}^{\prime },B,K\_AB\right\}K{}_{AS}. \end{array}$$

The intruder *I* pretends to be A and sends the intercepted encrypted message to the server *S* with the identifier of A;

(14) $$\begin{array}{c} S->B\text{:}\left\{T_{S}^{\prime \prime },A,K\_AB\right\}K{}_{BS}. \end{array}$$

*S* thinks that a new protocol session message is sent by *A*, so it updates the value of the timestamp and sends the message to *B*. After *B* receives the message, it determines that the timestamp is the latest, so *B* thinks that the key *K_AB* in the message is the latest.

But in fact, the key *K_AB* in the message is not the fresh key generated by the current round of the protocol, but the old key generated by the previous session. If *B* uses this key to encrypt important information and send it to *A*, agent *A* may think that the key has expired and refuse to receive it, which may cause adverse consequences.

Figure 12 shows the attack path of WMF protocol violating authentication (attack path 2); the attack path can be described as follows:

(15) $$\begin{array}{c} I\left(A\right)->S\text{:}A,\left\{T_{I},B,K\_IB\right\}K{}_{IS}. \end{array}$$

The intruder *I* generates the key *K_IB* and encrypts it with the timestamp *T*_*I*_ and the key receiving agent *B* with *K*_*IS*_ to form a ciphertext block. Immediately *I* attaches the agent identification of *A* to form a message and then sends it to *S*;

(16) $$\begin{array}{c} S->B\left\{T_{S},A,K\_IB\right\}K{}_{BS}. \end{array}$$

*S* determines that the message comes from *A* according to the identifier in the received message, so it updates the timestamp and encrypts it together with the agent identifier *A* and the key *K_IB to* form a message and then *S* sends the message to agent *B*. When receiving the message, *B* detects that the timestamp is the latest and then considers the message to be fresh. Finally, *B* determines that the key generator is *A* according to the identifier in the encrypted message.

But in fact, the agent *A* is faked by the intruder *I*; even the honest agent *A* does not participate in the operation of the protocol. If *B* encrypts important information with the received key *K_IB* and sends it to *A*, it will be intercepted and decrypted by an intruder *I* who pretends to be *A*. And if the intruder obtains the content of the message, there will be serious security problems.

## 7. Conclusion

The model checking technique and model checker SPIN are used to verify security protocols with timestamps and the model building method for security protocols with timestamps is introduced in this paper. We take the WMF protocol as an example to describe the modeling method in detail and the experimental results show that our method can successfully find the vulnerabilities of WMF protocol and there is no state space explosion problem. The results also show that our method is effective and it can provide a direction for the formal analysis of other security protocols with timestamp.

Since the time model represented by Promela in this paper is discrete and the time precision that can be represented is limited, the next work can be to try to use a more fine-grained time representation method to make our protocol model as identical as possible to the real-world protocol environment. In addition, another future work is to determine a unified principle for automatically finding out the vulnerable channels in protocols to help optimize the intruder's model.

## Figures and Tables

**Table 1:** Basic symbols and meanings.

Symbol	Meaning
*P*	Authentication and key agreement protocol
*Init*	Protocol initiator
*Rec*	Protocol responder
*Ser*	Trusted server
*sk*	Session key generated by the protocol initiator
*Ts*	The generation time of *sk*
*Tn*	The reception time of *sk*
*Dmax*	Maximum time spent in message transmission
*Dmin*	Minimum time spent in message transmission
*N*	The number of messages in a protocol

**Table 2:** Experimental data of WMF protocol.

WMF protocol model	Model 1 (using synchronization channel)		Model 2 (using asynchronous channels)	
Security properties	States	Transitions	States	Transitions
Authentication	323	398	361	448
Freshness	47	50	51	54

*Note.* The experimental results are obtained by using SPIN6.5.1, CPU Intel(R) Core (TM)i5-6300HQ (2.3 GHz), RAM 2 GB, and the operating system platform Ubuntu 18.04.6 virtual machine experimental environment. The parameter of the state search method is depth-first search, and the parameter value of the search depth is 10000 (default value).

**Table 3:** Experimental results of WMF protocol.

Verification results	Reference [22]	Reference [23]	This paper
Attack path 1	√	√	√
Attack path 2	—	—	√

## Data Availability

The data used to support the findings of this study are available from the corresponding author upon request.
